# Synthetic host defense peptide IDR-1002 reduces inflammation in *Pseudomonas aeruginosa* lung infection

**DOI:** 10.1371/journal.pone.0187565

**Published:** 2017-11-06

**Authors:** Kelli C. Wuerth, Reza Falsafi, Robert E. W. Hancock

**Affiliations:** Centre for Microbial Diseases and Immunity Research, Department of Microbiology and Immunology, University of British Columbia, Vancouver, British Columbia, Canada; Louisiana State University, UNITED STATES

## Abstract

*Pseudomonas aeruginosa* is a frequent cause of lung infections, particularly in chronic infections in cystic fibrosis patients. However, treatment is challenging due to *P*. *aeruginosa* evasion of the host immune system and the rise of antibiotic resistant strains. Host defense peptides (HDPs) and synthetic derivatives called innate defense regulators (IDRs) have shown promise in several infection models as an alternative to antibiotic treatment. Here we tested peptide IDR-1002 against *P*. *aeruginosa* in vitro and in vivo. Treatment of bronchial epithelial cells and macrophages with IDR-1002 or in combination with live *P*. *aeruginosa* or its LPS led to the reduction of agonist-induced cytokines and chemokines and limited cell killing by live *P*. *aeruginosa*. In an in vivo model using *P*. *aeruginosa* combined with alginate to mimic a chronic model, IDR-1002 did not reduce the bacterial burden in the lungs, but IDR-1002 mice showed a significant decrease in IL-6 in the lungs and in gross pathology of infection, while histology revealed that IDR-1002 treated mice had reduced alveolar macrophage infiltration around the site of infection and reduced inflammation. Overall, these results indicate that IDR-1002 has promise for combating *P*. *aeruginosa* lung infections and their resulting inflammation.

## Introduction

*P*. *aeruginosa* is commonly found in the environment but rarely causes infection in healthy individuals. However, in immunocompromised patients or when introduced into wounds, burns, or the urinary tract, *P*. *aeruginosa* can cause serious infections and even death [1–3]. Of particular concern are lung infections caused by *P*. *aeruginosa*, such as pneumonia or chronic infections in patients with cystic fibrosis (CF) or chronic obstructive pulmonary disease (COPD). *P*. *aeruginosa* is also one of the most frequent causes of nosocomial pneumonia in the ICU [2]. In CF and COPD, while initial infection might be similar to acute infections, over time the *P*. *aeruginosa* forms biofilms and undergoes a series of adaptations, including the decreased expression of flagella, thus leading to chronic infections. Over 80% of CF patients acquire *P*. *aeruginosa* by their mid-twenties, leading to increased hospitalizations and eventually death from loss of lung function [4, 5]. However, the treatment of *P*. *aeruginosa* lung infections is hampered by its inherent resistance to antibiotics as well as acquired and adaptive resistance mechanisms [6, 7]. Multi-drug resistant strains of *P*. *aeruginosa* are on the rise [8], and it has recently been classified as a serious threat by the U.S. Centers for Disease Control and as a critical threat by the World Health Organization due to the rise of multi-drug resistant strains of *P*. *aeruginosa* and a lack of new treatment options [9, 10]. Therefore, there is a need for alternatives to antibiotics for the treatment of *P*. *aeruginosa* lung infections.

HDPs, such as the human cathelicidin LL-37, are small, naturally occurring peptides that have shown profound immunomodulatory effects in vivo and under in vivo-like conditions, including a critical role in the host response to microbial infections [11]. These immunomodulatory effects prompt the host immune system to respond to microbial infections, thus limiting the potential development of antimicrobial resistance that occurs from direct targeting of the microbe as well as the negative effects of antibiotics on the host microflora. However, the use of HDPs as exogenous agents has been limited due to toxicity and the relatively expensive process of synthesis for these peptides due to length or the inclusion of more complex chemistry such as disulfide bonds [12, 13]. Therefore, synthetic versions have been developed with similar properties but with reduced size, toxicity, and cost [14–16]. While a handful of synthetic HDPs have been tested against *P*. *aeruginosa* lung infections in vivo, they have mostly been ineffective or shown toxic effects [17–19]. Some larger peptides (18-mers or longer) appeared to reduce colony-forming unit (CFU) burden in the lungs, but they were not evaluated for anti-inflammatory or other immunomodulatory effects [20]. Therefore, there is still a need for new peptides for the treatment of *P*. *aeruginosa* lung infections.

Synthetic HDPs developed in our laboratory, termed IDRs, are typically only 12 amino acids in length and have been effective in models of cerebral malaria, tuberculosis, and *S*. *aureus*-infected wounds and implants, and also have been effective against *P*. *aeruginosa* biofilms in vitro [21–25]. However, they have not been tested in vivo against *P*. *aeruginosa* infections. One IDR peptide, IDR-1002, has been tested in vivo against *S*. *aureus* and *E*. *coli* and shown anti-infective therapeutic efficacy [26]. These effects indicated that IDR-1002 might also be beneficial against *P*. *aeruginosa* infections.

In the present study, the potential of IDR-1002 as an anti-infective agent against *P*. *aeruginosa* lung infections was examined. First, IDR-1002 and the human HDP LL-37 were used in bronchial epithelial cells and macrophages, two key cell populations for the immune response during a lung infection, and their effects on toxicity and cytokine and chemokine release were evaluated alone or in combination with *P*. *aeruginosa* or its components. Next, a murine lung model was developed for testing IDR-1002. *P*. *aeruginosa* does not normally cause a chronic infection in mice, therefore the bacteria are often embedded in agar or agarose beads and then delivered intratracheally (IT) [27, 28]. Alternatively, a model was developed by Hoffmann *et al*. using a clinical isolate of *P*. *aeruginosa* mixed with alginate isolated from the same strain [29–31]. Alginate is an exopolysaccharide produced by *Pseudomonas* that is analogous to the alginate derived from seaweed. While both models give the *P*. *aeruginosa* some protection from the host immune system, using an actual biofilm matrix component, alginate, instead of agar better reflects the interactions of the host immune system with the bacteria during a chronic infection. To improve the throughput and make the model more representative of the typical route of lung infection, in this study alginate isolated from seaweed was used along with IN administration of the *P*. *aeruginosa* and alginate mixture. We also used the chronic epidemic CF patient isolate LESB58, which is now considered to be the first isolated Liverpool Epidemic Strain, a group of highly virulent strains that have increased antibiotic resistance and are associated with increased morbidity and transmission among CF patients [32–34]. LESB58 is a strong biofilm producer, and although it has a flagellum it shows decreased motility [35].

The results showed that IDR-1002 was effective against preventing *P*. *aeruginosa*-induced toxicity and could limit the release of pro-inflammatory cytokines. Additionally, while IDR-1002 did not affect the number of lung CFUs in the alginate model, it nevertheless decreased cytokines and damage in the lungs. Overall, the data indicate that IDR peptides could be novel therapeutic or prophylactic agents against *P*. *aeruginosa* lung infections.

## Materials and methods

### Mice and ethics statement

C57Bl/6J mice were ordered from Jackson Laboratory or were bred at the Modified Barrier Facility at the University of British Columbia. Female mice were used between 6–8 weeks of age. All experiments were approved by the UBC Animal Care Committee.

### Reagents

LL-37 (LLGDFFRKSKEKIGKEFKRIVQRIKDFLRNLVPRTES-NH_2_) was purchased from CPC Scientific (Sunnyvale, California, USA) and IDR-1002 (VQRWLIVWRIRK-NH_2_) was purchased from Kinexus (Vancouver, British Columbia, Canada). Both peptides were synthesized by F-moc chemistry and purified by HPLC to greater than 95% purity. Peptides were stored as desiccated powders at -20°C, then resuspended in endotoxin-free water for experiments and stored at -20°C unless otherwise noted. LPS from *P*. *aeruginosa* PAO1 (strain H103) was isolated in the laboratory by the Darveau-Hancock method, as previously described [36].

### Cell culture

Human bronchial epithelial cells, 16HBE14o- (HBE) cells, were grown in MEM with Earle’s salts (catalogue #11090, Thermo Fisher Scientific, Waltham, Massachusetts, USA) and mouse macrophage cells, RAW264.7 (RAW) cells, were grown in DMEM (catalogue #10313, Thermo Fisher Scientific), each with the addition of 10% (v/v) heat-inactivated fetal bovine serum (FBS) and 2 mM L-glutamine (Thermo Fisher Scientific). Cells were maintained at 37°C with 5% carbon dioxide and passaged by removing media, washing with PBS (Thermo Fisher Scientific), and adding 0.05% trypsin (Thermo Fisher Scientific) to detach cells. Cells were centrifuged, the supernatant was removed, and cells were resuspended and moved into new flasks.

For experiments, HBE cells were passaged into 48-well plates (1 x 10^5^ cells/ml, 0.5 ml per well) and allowed to grow for approximately two days to achieve greater than 80% confluency in a monolayer. At approximately 2 h before experiments, medium was removed, cells were washed with PBS, then fresh medium supplemented with L-glutamine and 1% FBS was added. Cells were rested for 2 h, then used for experiments. RAW cells were passaged into 48-well plates (1.5 x 10^5^ cells/ml, 0.5 ml per well) and allowed to grow overnight before being used for experiments. For treatments, samples received equivalent volumes of the diluents unless otherwise noted. Supernatants were collected and then stored at -20°C.

### Lactate dehydrogenase assay

Supernatants collected from cell cultures were combined at a 1:1 (v/v) ratio with complete lactate dehydrogenase (LDH) reagent (Roche Diagnostics, Basel, Switzerland). After incubating for 15–25 minutes in the dark, plates were read on a Power Wave X340 plate-reader (Bio-Tek Instruments, Winooski, Vermont, USA). Results were normalized to the control sample (0% toxicity) and a sample treated with 2% (v/v) Triton X-100 (100% toxicity).

### Bacterial culture

Bacterial strains *P*. *aeruginosa* PA103, LESB58, and PAO1 (strain H103) were streaked onto LB plates from frozen stocks and grown overnight at 37°C. The following day, individual CFUs were used to make overnight cultures in LB and grown overnight at 37°C with shaking. Overnight cultures were diluted 1:50 (PA103 and PAO1) or 1:10 (LESB58) and grown to an OD_600_ reading of approximately 0.5. Bacteria were then washed with endotoxin-free 0.9% sodium chloride saline, centrifuged, supernatant discarded, and the pellet resuspended in endotoxin-free saline to an OD_600_ of 0.5. Bacteria were then diluted to the appropriate concentration based on previous experiments relating OD_600_ reading and CFU/ml, and serial dilutions were plated on LB to check the final concentration.

### *Pseudomonas* lung infection model

*P*. *aeruginosa* PA103 or LESB58 was prepared as described above, except that for the alginate mouse model bacteria were resuspended in 11 mg/ml sodium alginate (Sigma-Aldrich) prepared in endotoxin-free saline. Mice were anesthetized with 2–5% isoflurane and placed on an intubation stand (BrainTree Scientific, Braintree, Massachusetts, USA). IDR-1002, *P*. *aeruginosa*, or appropriate controls were instilled dropwise using a micropipette into the left nostril of each mouse. *P*. *aeruginosa* was in a 20 μl volume, while peptide was approximately 10–20 μl depending on the weight of the mouse. Isoflurane was periodically applied to keep the mouse at a steady respiratory rate. After instillation, mice were kept on the stand under isoflurane for 2–3 minutes to ensure absorption of the liquid, then they were fully recovered and returned to their cages.

For sample collection, mice were euthanized with 120 mg/kg of IP injected sodium pentobarbital. Blood was collected from the inferior vena cava and allowed to clot, then centrifuged and serum collected for use in ELISAs. For lung homogenates, the lungs were collected and the lobes separated and placed in 300 μl of PBS, then homogenized on a Mini-Beadbeater-96 (BioSpec, Bartlesville, Oklahoma, USA) for 1 minute. The homogenates were filtered (40 μm filter, #431750, Corning) and the filtrate used for plating on LB as described above, then the remaining liquid was stored at -20°C for use in ELISAs.

For histology, a cannulated needle was used to deliver 4% paraformaldehyde (PFA) to the lungs, then the trachea was tied off with string to prevent leakage and the lungs were placed in 4% PFA. After two days, samples were washed with 70% ethanol. Wax-It Histology Services (Vancouver, British Columbia, Canada) embedded the samples in paraffin, sectioned them, and then stained slides with hematoxylin and eosin (H&E), periodic acid-Schiff (PAS), or Alcian blue. The slides were then evaluated and quantified by a trained pathologist.

### ELISAs

Samples were stored at -20°C until use in ELISAs. Levels of cytokines and chemokines were measured using eBioscience (San Diego, California, USA) antibodies for murine TNF-α and IL-6 and human IL-6. MCP-1 antibodies were from eBioscience or R&D Systems (Minneapolis, Minnesota, USA). KC (CXCL1) antibodies were from Fitzgerald (Acton, Massachusetts, USA) or R&D Systems. Human IL-8 antibodies were from Invitrogen (Waltham, Massachusetts, USA). Standards were purchased from the same sources. The ELISAs were performed by following the manufacturer protocols with optimization of antibody and sample dilutions, washes, and incubation times. They were developed using TMB (eBioscience) and the reaction stopped with 2 N sulfuric acid. The plates were read on a Power Wave X340 plate-reader (Bio-Tek Instruments) and fitted to a 4-parameter standard curve using KC4 software (Bio-Tek Instruments).

### Statistical analysis

Data were analyzed using Microsoft Excel 2013 and GraphPad Prism version 7. GraphPad Prism was used to perform an unpaired t-test, one-way ANOVA, or two-way ANOVA as needed. Tukey’s and Dunnett’s multiple comparisons tests were used when comparing the means of all samples or when comparing to only to a control sample, respectively. A repeated measures two-way ANOVA with Sidak’s multiple comparisons test was used to evaluate data taken over multiple time points (i.e., weights and health scores). A value of p ≤ 0.05 was considered statistically significant.

## Results

### IDR-1002 was not per se pro-inflammatory and it suppressed inflammatory cytokines induced by *P*. *aeruginosa* lipopolysaccharide

IDR-1002 (5 x 10^−4^ μM to 50 μM) or LL-37 (5 x 10^−4^ μM to 3 μM in HBE cells or 25 μM in RAW cells) was added to HBE cells or RAW cells at the same time as *P*. *aeruginosa* lipopolysaccharide (LPS; 10 ng/ml), then supernatants were collected after 24 h and used for ELISAs to measure the production of cytokines and chemokines in response to peptide alone or the combination of peptide and LPS. The maximum concentrations of LL-37 were selected based on the results of LDH cytotoxicity experiments (S1 Fig), with concentrations above 3 μM in HBE cells or 25 μM of LL-37 in RAW cells showing greater than 20% toxicity. In contrast, IDR-1002 treatment produced less than 5% toxicity in both HBE and RAW cells.

**Fig 1:**
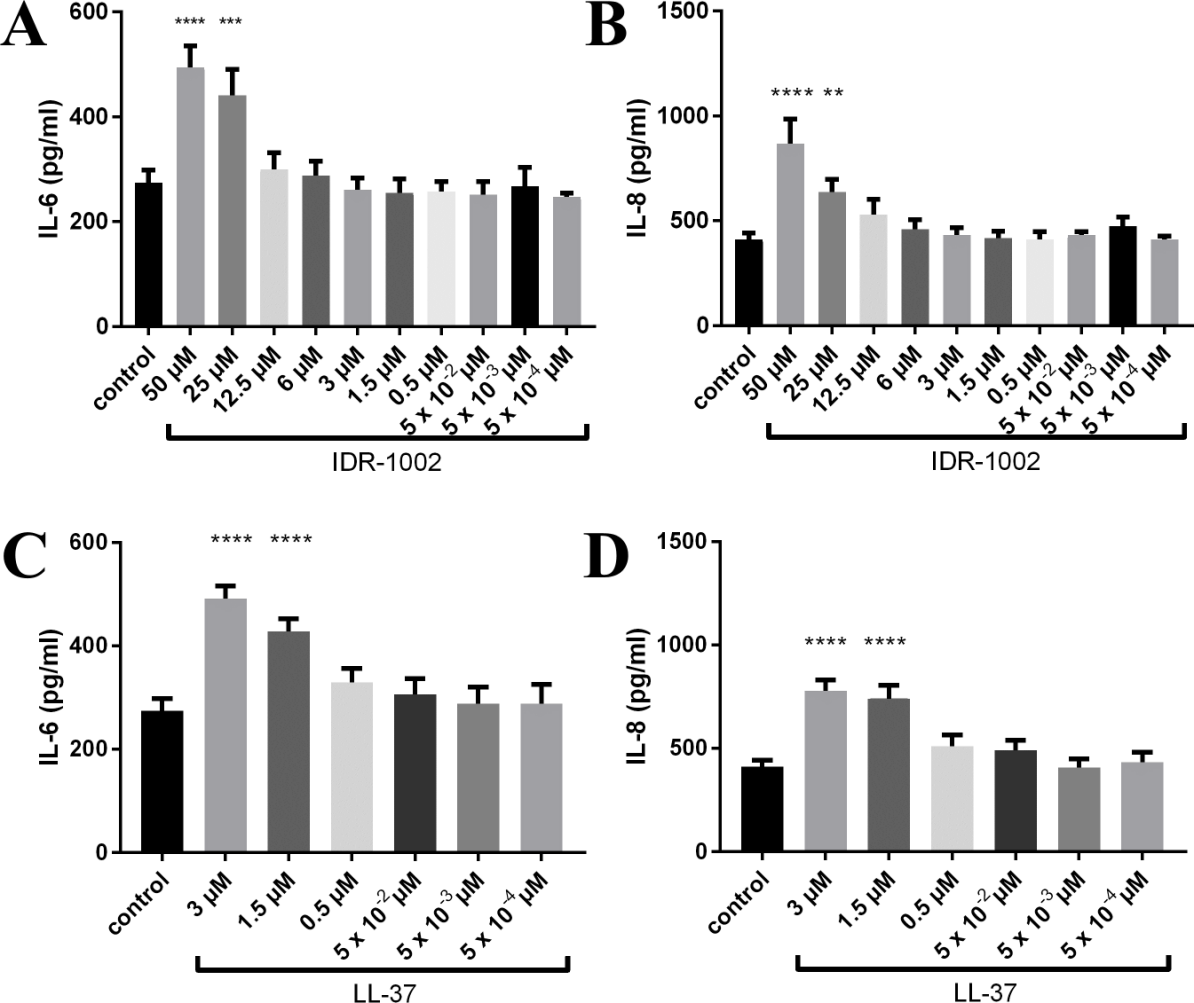
HBE cells showed increases in IL-6 and IL-8 in response to peptide. Peptide was added to the cells, then supernatants were collected after 24 h for ELISAs. IDR-1002 significantly increased IL-6 (A) and IL-8 (B), and LL-37 significantly increased IL-6 (C) and IL-8 (D). Data represent mean ± SEM from four independent experiments and were analyzed using two-way ANOVA and Dunnett’s multiple comparisons test. **: p ≤ 0.01, ***: p ≤ 0.001, ****: p ≤ 0.0001 compared to control.

In HBE cells, IL-6 and IL-8 production showed a dose-dependent increase in response to IDR-1002 or LL-37 alone (Fig 1). HBE cells did not demonstrate changes in IL-6 or IL-8 in response to LPS, but the combinations of IDR-1002 or LL-37 with LPS showed virtually identical results to those with peptide alone, with both peptides showing a dose-dependent increase (data not shown).

**Fig 2:**
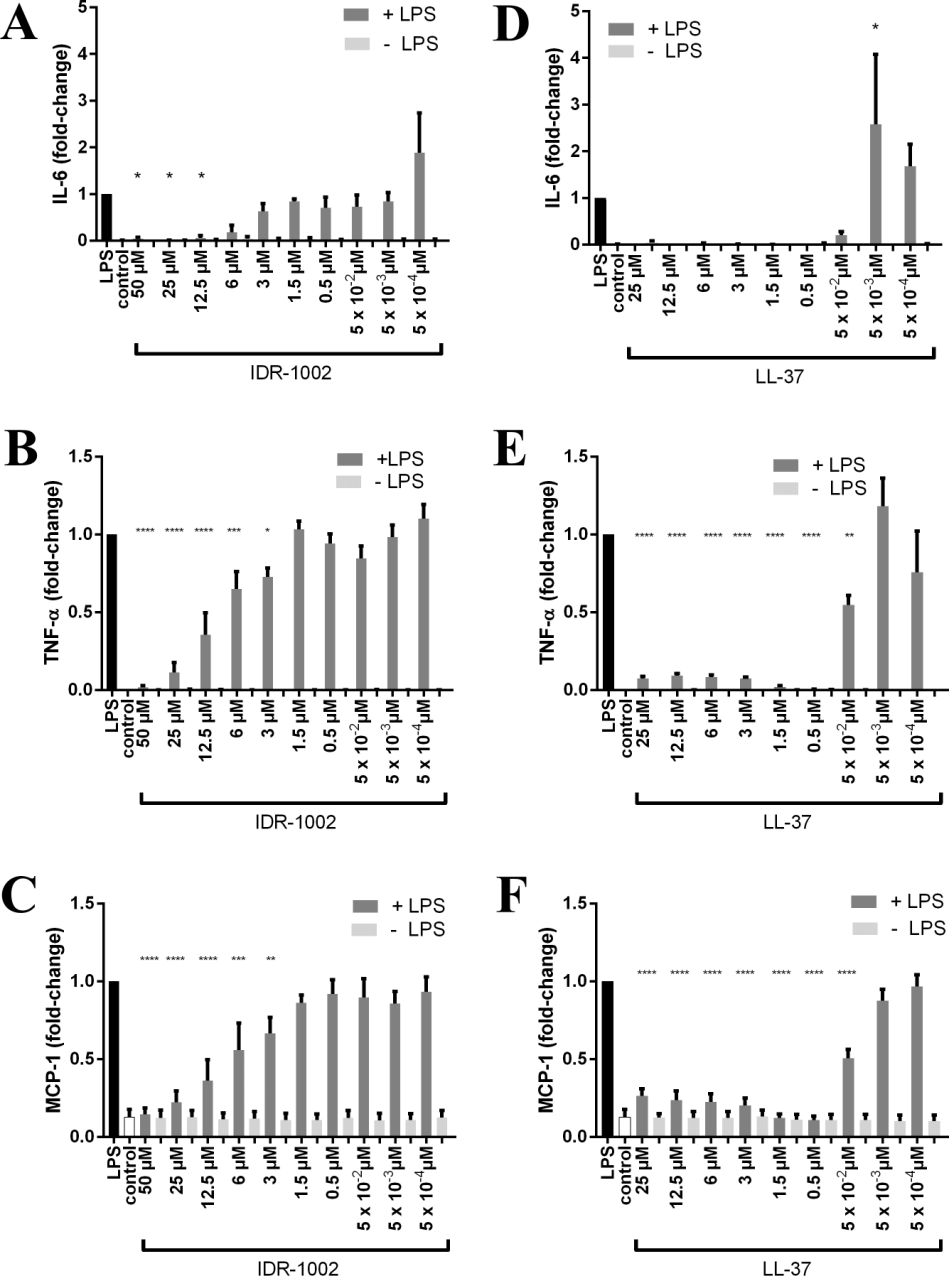
IDR-1002 and LL-37 reduced LPS-induced cytokines and chemokine MCP-1 in RAW cells. The listed concentrations of IDR-1002 or LL-37 were added with or without *P*. *aeruginosa* LPS (10 ng/ml), then supernatants were collected after 24 h. Without LPS, IDR-1002 and LL-37 did not induce any of the cytokines or chemokine. IDR-1002 decreased LPS-induced IL-6 (A), TNF-α (B), and MCP-1 (C). LL-37 also decreased LPS-induced IL-6 (D), TNF-α (E), and MCP-1 (F). Data represent mean ± SEM from four independent experiments expressed as fold-change relative to LPS and were analyzed using two-way ANOVA and Dunnett’s multiple comparisons test. Only the significance for samples given LPS is displayed. *: p ≤ 0.05, **: p ≤ 0.01, ***: p ≤ 0.001, ****: p ≤ 0.0001 compared to LPS.

In RAW cells, the levels of pro-inflammatory cytokines TNF-α and IL-6 in the supernatant were below the detectable limit for all doses of IDR-1002 and LL-37 (Fig 2). MCP-1 did not increase in response to either peptide. RAW cells showed significant increases in IL-6, TNF-α, and MCP-1 levels in response to LPS, but when higher doses of IDR-1002 or LL-37 were used in conjunction with LPS, the levels were returned to the baseline seen in the control without LPS. The decreases in cytokine or chemokine concentration were dose-dependent, with higher concentrations of peptide producing greater reductions in the LPS-induced response. However, lower concentrations of LL-37 actually increased the production of cytokines in combination with LPS, with IL-6 showing a significant increase compared to LPS alone.

**Fig 3:**
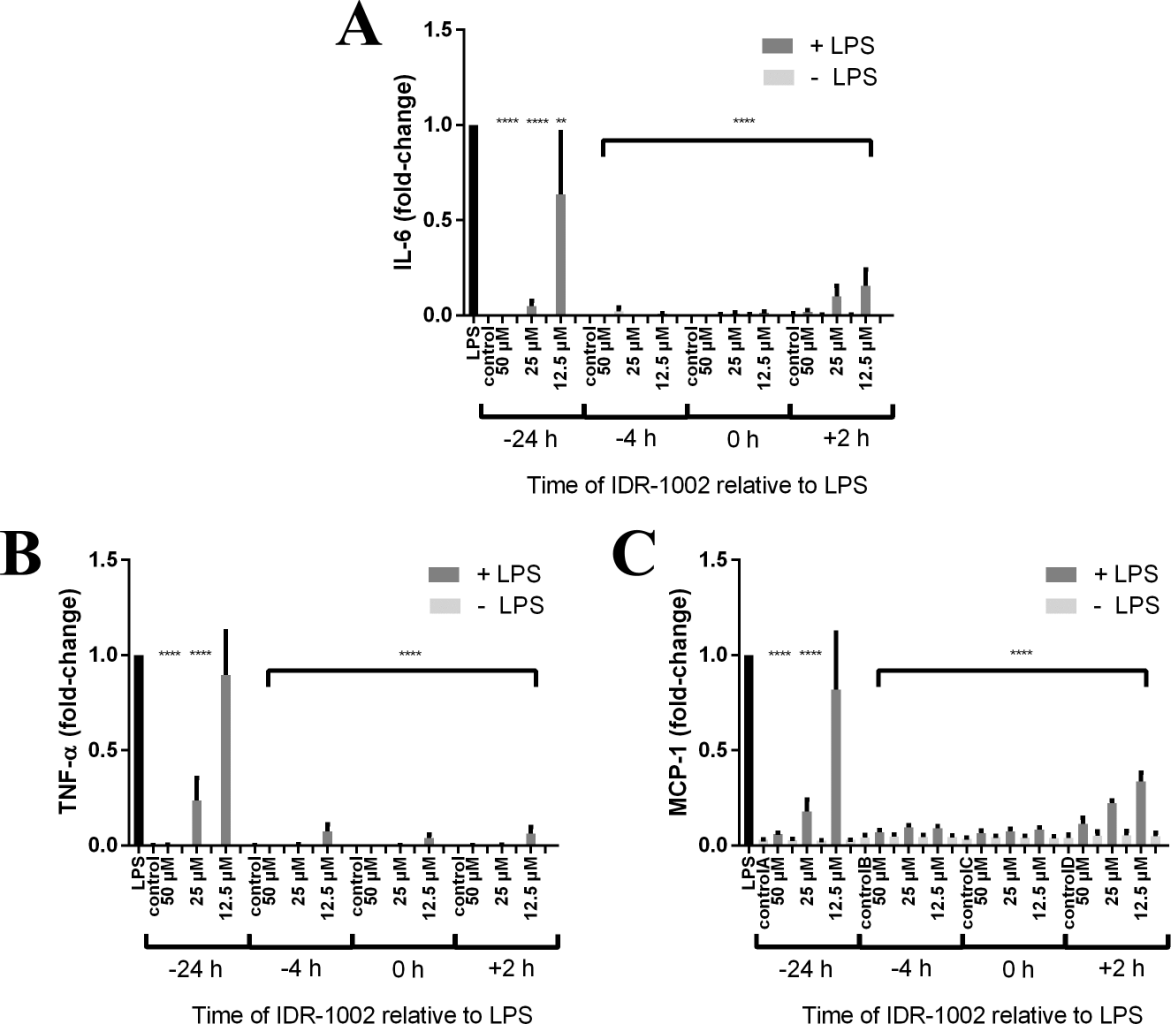
IDR-1002 reduced LPS-induced inflammatory cytokines and chemokine MCP-1 when added before or after the addition of LPS. IDR-1002 at 12.5, 25, or 50 μM was added 24 or 4 h prior to *P*. *aeruginosa* LPS, at the same time as LPS (0 h), or 2 hours after LPS. Supernatants were collected 24 h after the addition of LPS for use in ELISAs for IL-6 (A), TNF-α (B), and MCP-1 (C). Data represent mean ± SEM from three independent experiments expressed as fold-change relative to LPS at each time point and were analyzed using two-way ANOVA and Dunnett’s multiple comparisons test. Only the significance for samples given LPS is displayed. **: p ≤ 0.01, ****: p ≤ 0.0001 compared to LPS.

To further examine the effects of IDR-1002 on LPS exposure, RAW cells were treated with 12.5, 25, or 50 μM IDR-1002 at 24 or 4 hours prior to LPS exposure, 2 hours after LPS exposure, or at the same time as LPS as in the previous experiment, with each time point also having an LPS control and a control without LPS. Samples were collected 24 h after LPS addition and used in ELISAs (Fig 3). Intriguingly, IDR-1002 significantly reduced IL-6, TNF-α, and MCP-1 at all times points of application relative to LPS treatment and at most concentrations, with the exceptions being 12.5 μM IDR-1002 given 24 h prior to LPS for MCP-1 and TNF-α. Even when given 2 h after the addition of LPS, IDR-1002 still reduced IL-6, TNF-α, and MCP-1 in a dose-dependent manner with less than half of the cytokines and chemokine produced relative to that due to LPS alone. This demonstrated that IDR-1002 has the potential to be effective as a prophylactic or therapeutic agent.

### IDR-1002 reduced toxicity caused by *P*. *aeruginosa* in vitro

**Fig 4:**
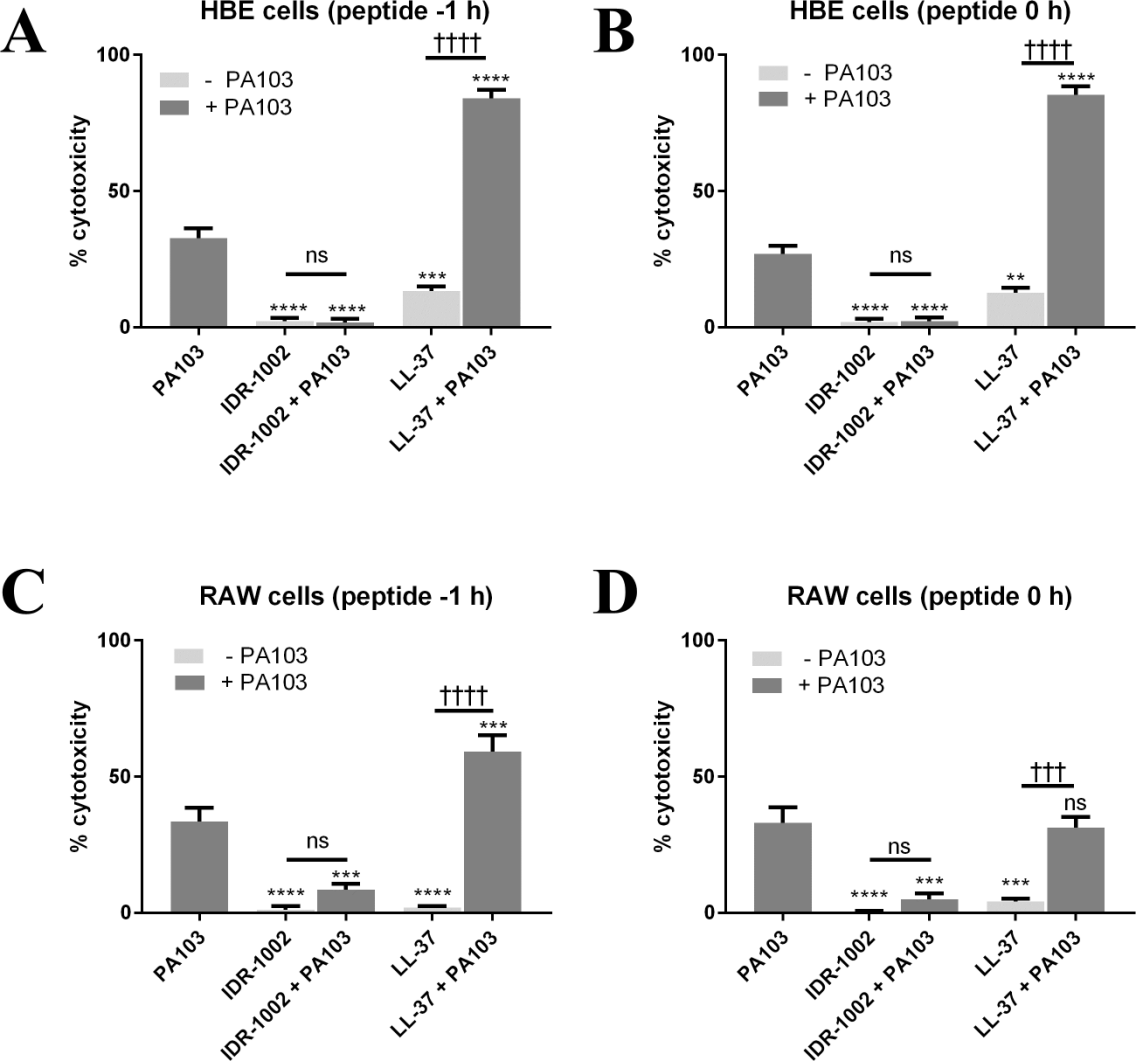
IDR-1002 limited cytotoxicity of *P*. *aeruginosa* in HBE and RAW cells, whereas LL-37 increased it. (A, B) Live *P*. *aeruginosa* PA103 (MOI 4) was added to HBE cells at 0 h, IDR-1002 (50 μM) or LL-37 (3 μM) was added at -1 h (A) or 0 h (B), then samples were collected at 6 h. (C, D) Live *P*. *aeruginosa* PA103 (MOI 1) was added to RAW cells at 0 h, IDR-1002 (50 μM) or LL-37 (12.5 μM) was added at -1 h (C) or 0 h (D), then samples were collected at 4 h. For both RAW and HBE cells, Triton X-100 was added at 0 h and samples were compared to a control (0% toxicity) and Triton X-100 (100% toxicity) for each time point. Data represent mean ± SEM from four independent experiments and were analyzed using two-way ANOVA and Tukey’s multiple comparisons test. *: p ≤ 0.05, **: p ≤ 0.01, ***: p ≤ 0.001, ****: p ≤ 0.0001 when compared to PA103. †††: p ≤ 0.001, ††††: p ≤ 0.0001 when samples compared as indicated in the figure.

While bacterial components such as LPS can cause inflammation, it is also important to examine the effects of living bacteria. After determining the best multiplicity of infection (MOI) and timepoint for RAW and HBE cells (S2 Fig), the effects of peptides on the live *P*. *aeruginosa* PA103 were tested. In HBE cells, PA103 (MOI 4) was added at 0 h, and either IDR-1002 (50 μM) or LL-37 (3 μM) was added to the wells at -1 h or 0 h in relation to infection with PA103. Triton X-100 was also added at 0 h. Samples were collected at 6 h post-infection and used for LDH cytotoxicity assays and ELISAs. For RAW cells, the design was similar except that strain PA103 was used at an MOI of 1, LL-37 was used at 12.5 μM, and all samples were collected at 4 h post-infection. In HBE cells, IDR-1002 blocked the cytotoxic effects of PA103, instead showing cytotoxicity levels near those of the control or IDR-1002 by itself (Fig 4A and 4B). The combination of LL-37 and PA103 induced a synergistic effect in increasing cytotoxicity as opposed to reducing the toxicity or simply having an additive effect.

In RAW cells, IDR-1002 showed a similar blocking effect for PA103 cytotoxicity, but the combination of PA103 and LL-37 showed an increase in toxicity compared to PA103 alone when the peptide was delivered at -1 h (Fig 4C). However, when LL-37 was delivered at the same time as PA103 in RAW cells, the toxicity of the combination was similar to PA103 alone (Fig 4D).

**Fig 5:**
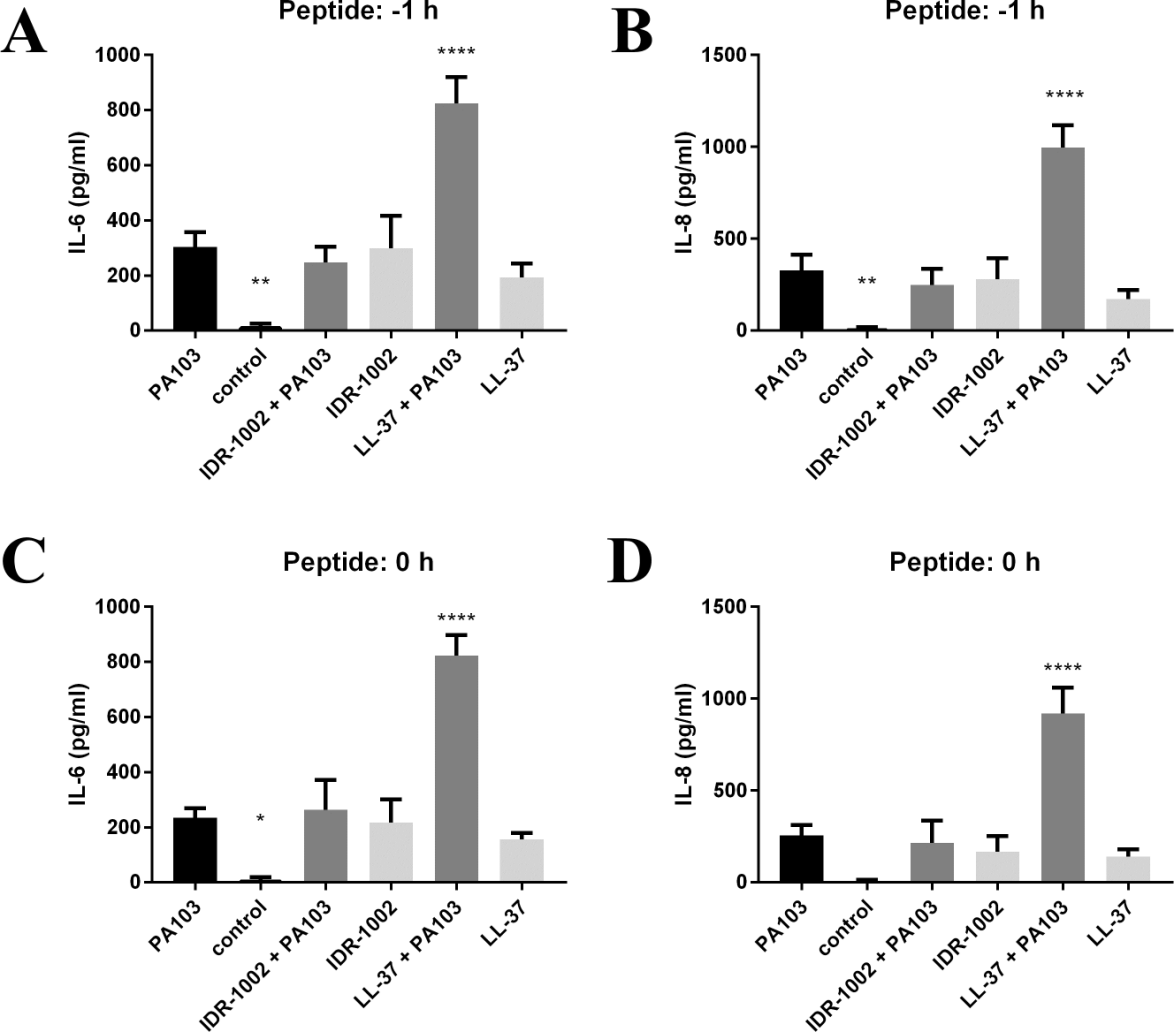
IL-6 and IL-8 production in HBE cells in response to live *P*. *aeruginosa* PA103. PA103 (MOI 4) was added at 0 h, then IDR-1002 (50 μM) or LL-37 (3 μM) was added at -1 h (A, B) or 0 h (C, D). Samples were collected at 6 h and used for ELISAs for IL-6 (A, C) or IL-8 (B, D). Data represent mean ± SEM from four independent experiments and were analyzed using two-way ANOVA and Dunnett’s multiple comparisons test. *: p ≤ 0.05, **: p ≤ 0.01, ****: p ≤ 0.0001 compared to PA103.

Cytokine and chemokine levels in the supernatants were also examined. In HBE cells, IL-6 and IL-8 showed slight increases when either peptide or PA103 was added, but the combination of LL-37 and PA103 showed significant increases in both markers (Fig 5). IL-6 and TNF-α were below the detectable limit in RAW cell samples, while MCP-1 concentrations were mostly undetectable and all were below 200 pg/ml, whereas the use of LPS for 24 h induced approximately 10 ng/ml of MCP-1.

### IDR-1002 reduced lesion sizes and lung damage in the alginate model

**Fig 6:**
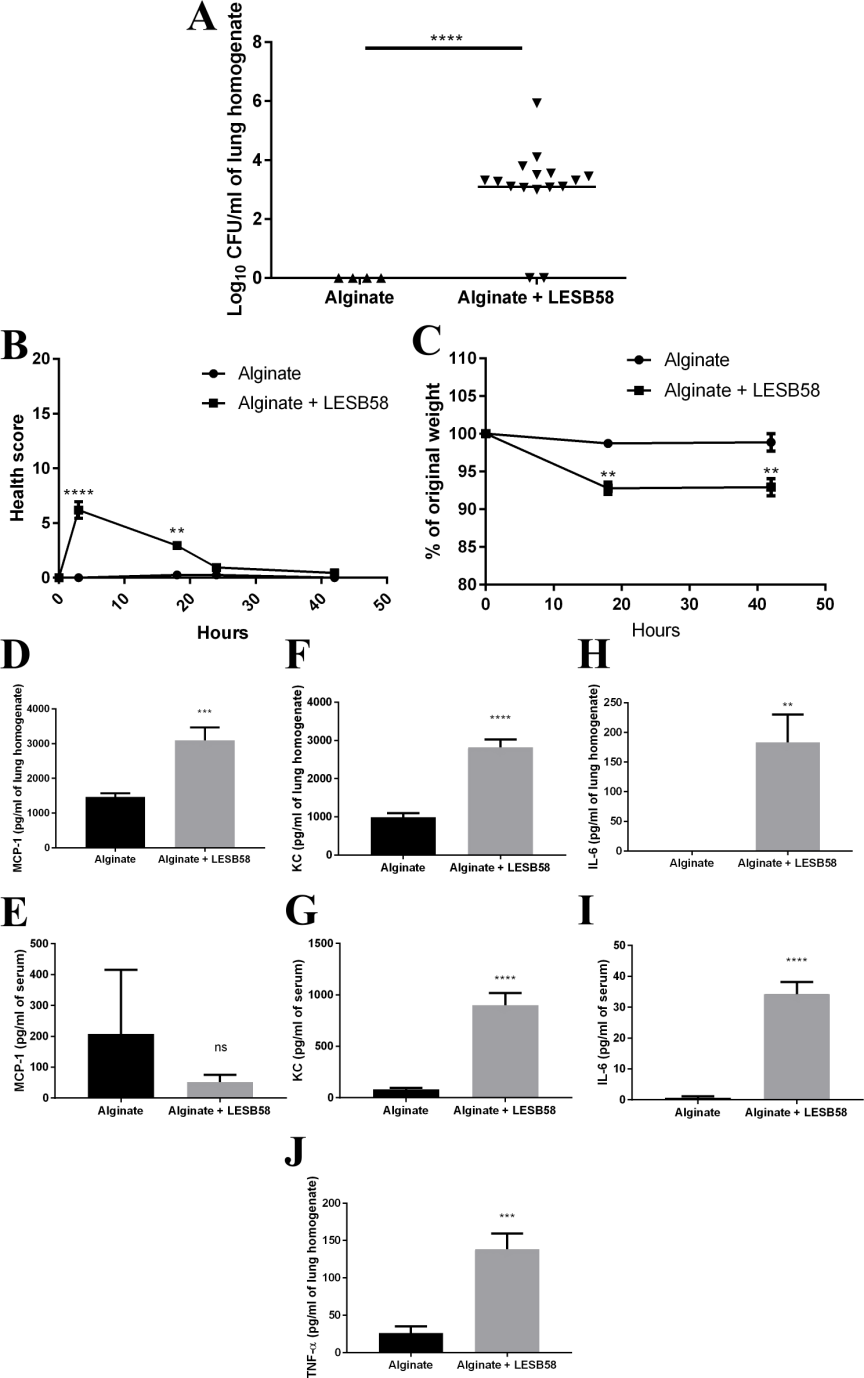
*P*. *aeruginosa* LESB58 was present two days post-infection when delivered with alginate. Mice were given alginate or alginate mixed with *P*. *aeruginosa* LESB58 (7 x 10^6^ CFU/mouse) IN at 0 h, then euthanized and samples processed at 42 h. (A) CFU counts from the lung homogenate. (B) Health scores over the course of the experiment. (C) Percentage of original weight for mice over the course of the experiment. ELISAs were performed for MCP-1 in lung homogenate (D) and serum (E); KC in lung homogenate (F) and serum (G); IL-6 in lung homogenate (H) and serum (I); and TNF-α in lung homogenate (J). Data represent mean ± SEM for n = 4 or 16 mice per condition from the combination of two experiments and were analyzed using unpaired t-test with Welch’s correction for 6A and 6D-6J, and with a repeated measures two-way ANOVA with Sidak’s multiple comparison for 6B and 6C. **: p ≤ 0.01, ***: p ≤ 0.001, ****: p ≤ 0.0001.

IDR-1002 showed encouraging results prophylactically against *P*. *aeruginosa* PA103 in an acute lung model by decreasing CFU burden and inflammatory mediators in the bronchoalveolar lavage fluid (BALF) and serum (S3 Fig). However, the model was not conducive for testing IDR-1002 therapeutically because the mice became sick rapidly (within two hours) and visible signs of infection typically increased over the course of the experiment, making it difficult to humanely anesthetize the mice and deliver IDR-1002 IN even at the early stages of infection. Decreasing the number of bacteria delivered in order to extend the model to multiple days was also ineffective, as the mice simply cleared the bacteria and appeared fully recovered a few hours after infection. Therefore, a lung model was developed based on the work of Hoffmann *et al*. [29], who used alginate isolated from a clinical strain of *P*. *aeruginosa*, then mixed the alginate with the same strain and delivered it IT. In our investigation, we used seaweed alginate to provide consistency and simplicity. It was mixed with the clinical isolate strain *P*. *aeruginosa* LESB58, as initial experiments using PA103 did not produce a sustained infection, and the mixture was then delivered IN, which is faster and less invasive than IT. Each mouse received 7 x 10^6^ CFU delivered in a 20 μl volume at 0 h. The mice were monitored at 3, 18, 24, and 42 h post-infection and evaluated for relative temperature, appearance of fur and eyes, activity levels, hunching, and respiratory effort. The mice were euthanized on day 2 at approximately 42 h post-infection. The lungs were homogenized and plated for CFUs. As expected, the mice given only alginate did not have *P*. *aeruginosa* in their lungs, while the mice given alginate mixed with *P*. *aeruginosa* LESB58 had CFUs recovered from their lungs (Fig 6A). Before homogenization, the lungs of alginate + LESB58 mice also appeared to demonstrate redness around the opening of the trachea into the lungs, which was absent from the mice given only alginate. The alginate + LESB58 mice showed a slight initial worsening of health scores (increased numbers) but recovered by 24 h, although they lost about 6–7% of their initial weight by 18 h after infection and this was maintained throughout the course of the experiment (Fig 6B and 6C). The lung homogenate and serum were used for ELISAs (Fig 6D–6J). Despite the fact that measurements occurred two days after the initial infection, the alginate + LESB58 mice had significant increases in MCP-1, KC, IL-6, and TNF-α in the lungs and in KC and IL-6 in the serum. TNF-α in the serum was below the detectable limit.

**Fig 7:**
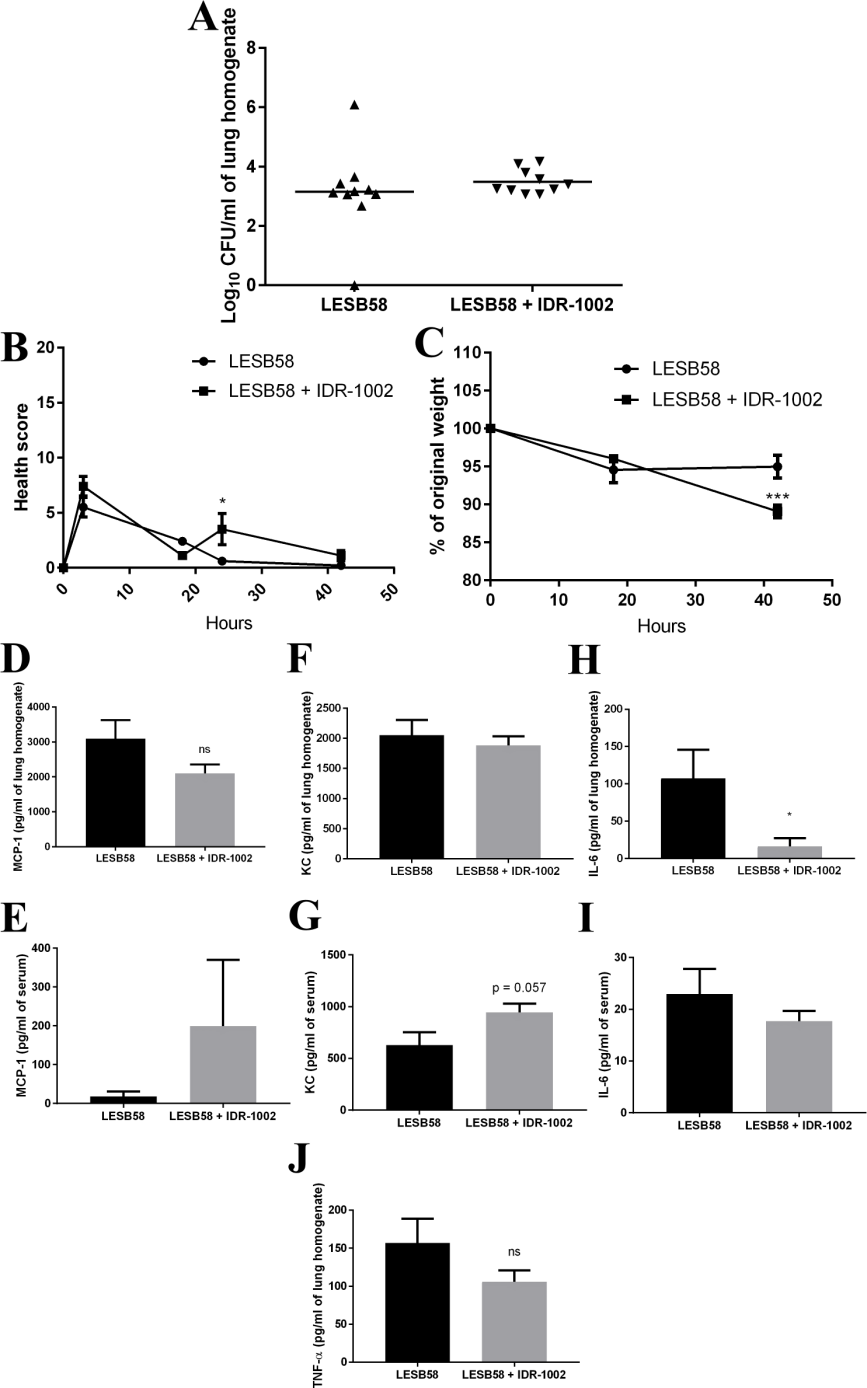
IDR-1002 decreased cytokines and chemokines in the alginate model. Mice were given alginate mixed with *P*. *aeruginosa* LESB58 (7 x 10^6^ CFU/mouse) IN at 0 h, then IDR-1002 (12 mg/kg) or vehicle-control were given IN at 18 h post-infection, then euthanized and samples processed at 42 h. (A) CFU counts from the lung homogenate. (B) Health scores over the course of the experiment. (C) Percentage of original weight for mice over the course of the experiment. ELISAs were performed for MCP-1 in lung homogenate (D) and serum (E); KC in lung homogenate (F) and serum (G); IL-6 in lung homogenate (H) and serum (I); and TNF-α in lung homogenate (J). Data represent mean ± SEM for n = 9 or 10 mice per condition from the combination of two experiments and were analyzed using unpaired t-test for 7A and 7D-7J, and with a repeated measures two-way ANOVA with Sidak’s multiple comparison for 7B and 7C. *: p ≤ 0.05.

Mice were given 7 x 10^6^ CFUs of *P*. *aeruginosa* LESB58 in alginate IN at 0 h, then at 18 h post-infection mice were given IDR-1002 (resuspended in endotoxin-free water) at 12 mg/kg IN while vehicle-control infected mice received an equivalent volume of endotoxin-free water IN. Endotoxin-free water was used for the control mice (rather than a scrambled peptide) because peptides demonstrate a broad range of activities and it is difficult, if not impossible, to conclude that a scrambled peptide is truly a negative control with no effects in vivo. Additionally, the use of scrambled peptides relates to the historical concept of distinct HDP and receptor interactions, but it now appears likely that HDPs bind to anionic surfaces rather than discrete binding sites [37, 38]. Mice were then euthanized at 42 h and the lungs homogenized and plated for CFUs. The number of recovered CFUs was indistinguishable between the two groups (Fig 7A). Mice actually showed a slight, but significant, worsening of health scores 6 h after being treated with IDR-1002 (i.e., at the 24 h time point) when compared to the vehicle-control mice given endotoxin-free water, although all mice including those given IDR-1002 recovered by 42 h (Fig 7B). The IDR-1002 treated mice also demonstrated an 11% decrease in weight by 42 h, while the control mice only had a 5.5% decrease (Fig 7C). The lung homogenate and serum were also used in ELISAs (Fig 7D–7J). In the IDR-1002 treated mice, IL-6 in the lung homogenate was significantly decreased, while the MCP-1 and TNF-α also trended towards showing decreases.

**Fig 8:**
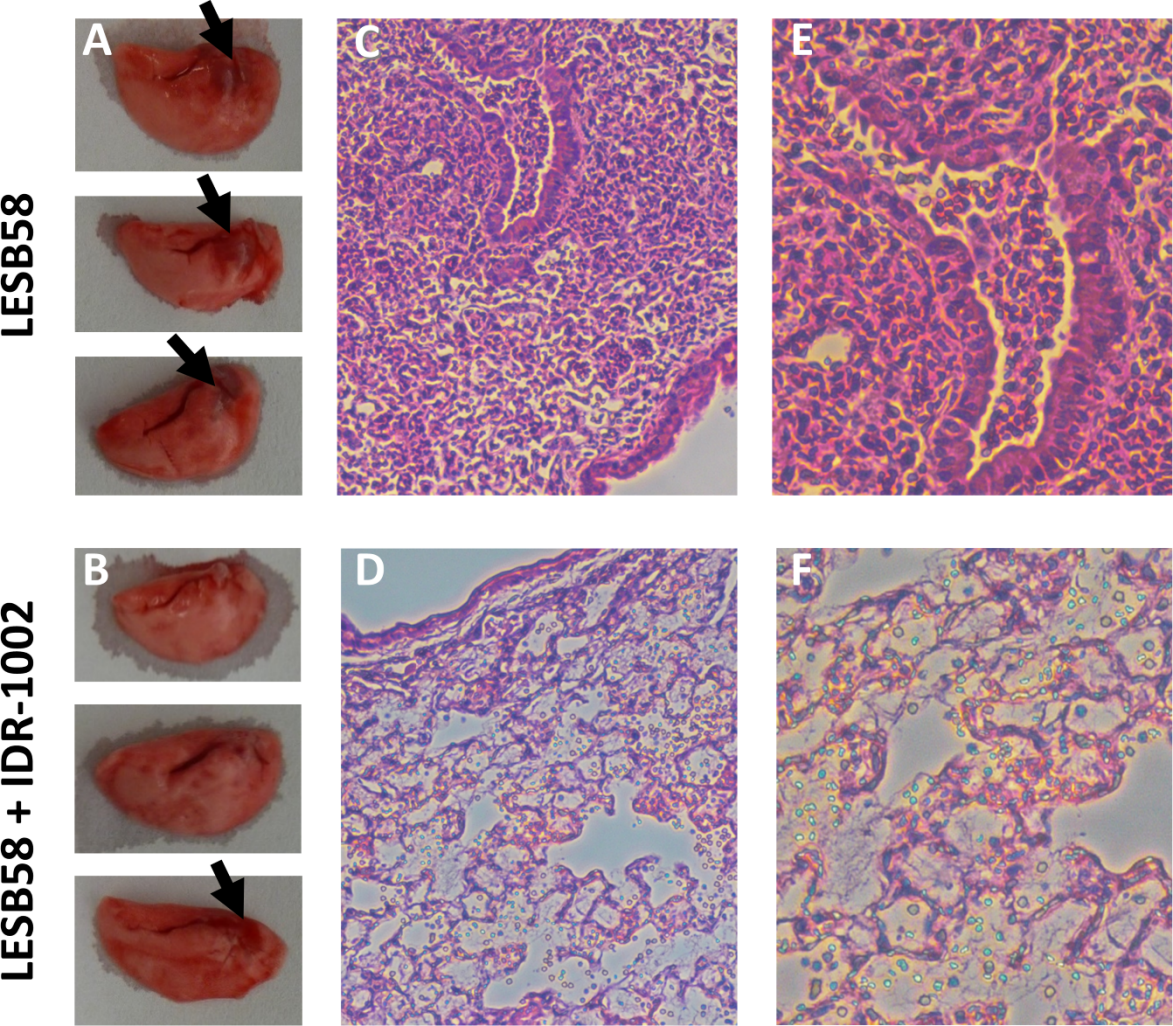
IDR-1002 mice demonstrated improved lung appearance in the alginate model. Mice were given alginate mixed with *P*. *aeruginosa* LESB58 (7 x 106 CFU/mouse) IN at 0 h, then IDR-1002 (12 mg/kg) or control IN at 18 h, then euthanized at 42 h. Vehicle control infected mice (A) demonstrated lesions (black arrows), whereas in the IDR-1002 mice (B) the lesions were reduced or eliminated. H&E staining in the control mice (C) showed damage, while in IDR-1002 mice (D) it was reduced. This was also seen with H&E staining in control mice (E) or IDR-1002 mice (F) at 20x.

Vehicle-control mice showed areas of redness near the opening to the trachea (Fig 8). However, the IDR-1002 treated mice either did not show these lesions or they were greatly reduced. The histopathological results were quantified in a blinded fashion by a trained pathologist (Table 1). Interestingly, while the neutrophil infiltration was similar for both LESB58 and LESB58 + IDR-1002 mice, the mice treated with IDR-1002 showed a decrease in alveolar macrophages at the infection sites. The IDR-1002 treated mice also had reductions both in the percentage of inflammation by area and in the number of mice with airway inflammation. No major differences were seen between vehicle control mice and IDR-1002 mice in the PAS or Alcian blue stained samples (S4 Fig).

**Table 1:** IDR-1002 mice showed decreased inflammation and alveolar macrophages in assessment of histopathology in the alginate model.

Condition	Histopathology Assessment			
	Neutrophilic inflammation (alveolar/ interstitial)	Alveolar macrophages in the area of acute inflammation	Inflammation in airways	Percentage of the section area with inflammation
LESB58	3.0 ± 0.0	2.2 ± 0.37	0.8 ± 0.20	19% ± 2.9
LESB58 + IDR-1002	2.6 ± 0.4	0.8 ± 0.37	0.4 ± 0.24	14.4% ± 2.6

Mice were given alginate mixed with *P*. *aeruginosa* LESB58 (7 x 106 CFU/mouse) IN at 0 h, then IDR-1002 (12 mg/kg) or control IN at 18 h, then euthanized at 42 h. Sections of lung were scored for neutrophilic inflammation and alveolar macrophages on a scale of 0–3 with no inflammation/infiltrate = 0, mild = 1, moderate = 2 and severe = 3. Inflammation in airways was assessed as no = 0 and yes = 1. Data represent mean ± SEM for n = 5 mice per condition from one experiment.

## Discussion

In both in vitro and in vivo models of *P*. *aeruginosa* lung infection, IDR-1002 demonstrated an ability to reduce inflammatory mediators such as TNF-α or IL-6 that are associated with microbial infections. Many *P*. *aeruginosa* lung infections, such as the chronic infections seen in CF, are highly inflammatory, and so the ability to target both *P*. *aeruginosa* and the associated inflammatory response would be highly beneficial. Importantly, IDR-1002 did not demonstrate any toxicity, whereas natural human HDP LL-37 caused toxicity at higher concentrations in vitro. In RAW cells, IDR-1002 did not induce TNF-α or IL-6, while in HBE cells increases in IL-6 were slight when compared to using similar concentrations of LL-37. IDR-1002 also did not show any toxicity in vivo in the acute *P*. *aeruginosa* model, and while mice used in the alginate model were slow to recover and had similar or slightly worse health scores soon after IDR-1002 delivery, their health scores had returned to baseline within a few hours.

IDR-1002 also did not increase MCP-1 levels in RAW macrophages. This is in contrast to previous results with human peripheral blood mononuclear cells (PBMCs) and mouse peritoneal lavage cells, where IDR-1002 strongly induced MCP-1 [21, 26]. However, in agreement with our results, in mouse bone marrow-derived macrophages MCP-1 mRNA was not significantly upregulated at four hours after IDR-1002 exposure [26]. PBMCs contain T cells, B cells, and monocytes, with small numbers of DCs and natural killer cells, while peritoneal lavage mostly contains B cells, T cells, and naïve macrophages [39]. It is possible that the induction of MCP-1 in PBMCs and peritoneal lavage cells is due to its expression by a small population of cells, such as γδ T cells [40], or that it requires the interactions of particular combinations of cell types. Another factor might be the cellular differentiation state, as RAW cells and BMDMs are differentiated macrophages, whereas PBMCs contain monocytes and peritoneal lavage often contains naïve macrophages. IDR-1002 did however increase a different chemokine, IL-8, in the HBE cells, but since these cells do not express MCP-1, its expression could not be examined. Further research into the induction of MCP-1 by IDR-1002 in specific cell populations would provide insights into the mechanisms of this response.

In RAW cells, *P*. *aeruginosa* LPS increased the expression of IL-6, TNF-α, and MCP-1, but in the presence of IDR-1002 these mediators were significantly reduced, even when IDR-1002 was added up to 24 h prior to, or 2 h after, the addition of LPS. LPS uptake is substantially complete within an hour in mouse monocytic cells [41], indicating that IDR-1002 inhibition of LPS responses is likely due to effects on signaling rather than direct interaction with LPS. Consistent with this, it was reported that IDR-1002 added 1 h prior to LPS stimulation in RAW cells limited the translocation of NF-κB to the nucleus by inhibiting the phosphorylation of the NF-κB translocation inhibitor, IκBα, and this mechanism might also be involved in the results seen here [42].

In the presence of live *P*. *aeruginosa* in RAW cells, LL-37 did not decrease its toxic effects, and actually produced a slight increase in *P*. *aeruginosa* PA103 toxicity when added one hour prior to bacteria. In contrast, IDR-1002 caused a significant reduction in cytotoxicity almost to baseline. In HBE cells, IDR-1002 blocked the cytotoxic effects of PA103, instead showing cytotoxicity levels near those of the control or IDR-1002-treated cells, while LL-37 with PA103 led to a greater than two-fold synergistic increase in cytotoxicity. The combination of LL-37 and PA103 also significantly increased IL-6 and IL-8 expression by nearly three-fold, whereas IDR-1002 showed no synergistic effect. The synergy of LL-37 and *P*. *aeruginosa* PAO1 in inducing cell death in both HBE cells and primary airway epithelial cells has been previously reported and found to be caused by the induction of apoptosis [43]. The effects of IDR-1002 in basically eliminating PA103-induced cytotoxicity might have been due in part to direct antimicrobial killing based on published MIC values [44], although tissue culture medium strongly antagonizes direct antimicrobial activity, or could reflect the lack of effect of this peptide on apoptotic cell death.

The alginate model showed a sustained infection over two days, with infected mice displaying a slight initial increase in health scores within 3 h but completely recovering by 24 h, although they maintained slight weight losses for 42 h. Importantly, significant increases were still seen in inflammatory cytokines in both the lungs and the serum of infected mice after two days when compared to the mice given only alginate. Additional testing of the model, such as extending it to longer time points, will help confirm the model as a prototype for testing agents in chronic *P*. *aeruginosa* lung infections, such as those seen in CF. When therapeutic IDR-1002 was given at 18 h post-infection, no changes were seen in the CFU burden. However, the peptide-treated mice had a significant reduction in IL-6 concentrations in the lung homogenate, and the macroscopically visible lesions were either eliminated or greatly reduced, consistent with the in vitro properties of this peptide. Histology revealed that IDR-1002 treatment reduced alveolar macrophage infiltration compared to the vehicle control infected mice. This was consistent with the anti-inflammatory effect of IN-delivered IDR-1002 in the acute *P*. *aeruginosa* lung infection model (S3 Fig), which also led to significantly reduced bacterial counts at the highest peptide dose. In contrast, IP-delivered IDR-1002 in the acute model was completely ineffective, with no changes in the CFU burden or lung appearance and no reduction in cytokines or chemokines in the BALF or serum, possibly indicating that IDR-1002 needs to be delivered near the site of infection in order to exert its effects (S5 Fig). In the alginate model, decreases in lung cytokine concentrations and the differences in the lung pathology indicated that, despite the lack of change in the CFU counts, IDR-1002 still seemed to be promoting an anti-inflammatory immune response against the infection. It is possible that mixing bacteria with alginate reduced the access of phagocytic cells to these bacteria, and given that phagocytes appear to be highly influential in protection by IDR peptides [26, 45], this might have impeded the immune response induced by IDR-1002.

In conclusion, this research showed the potential of IDR-1002 as a treatment of *P*. *aeruginosa* lung infections. IDR-1002 reduced pro-inflammatory cytokines in vitro and in vivo. As excessive inflammation is a life-threatening feature in chronic *P*. *aeruginosa* lung infections, the results are encouraging for the adjunctive use of IDR-1002 against *P*. *aeruginosa* infections. Critically, IDR-1002 demonstrated both effectiveness and safety, two features that were limited in previous peptide-based drugs against *P*. *aeruginosa* in vivo lung infections [17–20]. Overall, IDR-1002 shows promise as a new agent to combat *P*. *aeruginosa* lung infections.

## Supporting information

S1 Fig IDR-1002 was not toxic in HBE cells or RAW cells, while LL-37 demonstrated cytotoxicity. The listed concentrations of IDR-1002 (A, B) or LL-37 (C, D) were added to cells, then supernatants were collected after 24 h and used in an LDH cytotoxicity assay. The horizontal line represents 20% cytotoxicity. Data represent mean ± SEM from four (HBE cells) or five (RAW cells) independent experiments.
(TIF)

S2 Fig *P*. *aeruginosa* caused toxicity in HBE cells and RAW cells. Live *P*. *aeruginosa* PA103 at MOIs of 0.5, 1, 2, or 4 was added to HBE cells (A) or RAW cells (B) at time 0 h at the same time as control and Triton X-100 samples. Samples were collected at 2, 4, or 6 h and compared to a control and Triton X-100 for each time point in an LDH cytotoxicity assay.
(TIF)

S3 Fig Intranasal IDR-1002 reduced *P*. *aeruginosa* CFUs in the BALF and cytokines and chemokines in the BALF and serum. Mice were given water or IDR-1002 (4, 6, or 8 mg/kg) IN at -24 h, given 8 x 10^5^ CFUs of *P*. *aeruginosa* PA103 IN at 0 h, then euthanized at 18 h. (A) CFU counts from the BALF. ELISAs were performed for MCP-1 in BALF (B) and serum (C); KC in BALF (D) and serum (E); IL-6 in BALF (F) and serum (G); and TNF-α in BALF (H). Data represent n = 3 mice per condition from one experiment and were analyzed using one-way ANOVA and Dunnett’s multiple comparisons test. *: p ≤ 0.05, **: p ≤ 0.01, ***: p ≤ 0.001.
(TIF)

S4 Fig No differences were observed in PAS or Alcian blue staining for control and IDR-1002 mice. Mice were given alginate mixed with *P*. *aeruginosa* LESB58 (7 x 106 CFU/mouse) IN at 0 h, then IDR-1002 (12 mg/kg) or control IN at 18 h, then euthanized at 42 h. PAS staining in the control mice (A) and IDR-1002 mice (B) and Alcian blue staining in the control mice (C) and IDR-1002 mice (D) appeared similar between the two groups.
(TIF)

S5 Fig IDR-1002 delivered intraperitoneally (IP) did not change CFU burden, total leukocytes in the BALF, or signs of infection. Mice were injected IP with saline or IDR-1002 (2 or 4 mg/kg) at -4 h, instilled IN with 4 x 10^5^ CFUs of *P*. *aeruginosa* PA103 at 0 h, then euthanized and samples processed at 18 h. (A) CFU counts from the BALF. ELISAs were performed for MCP-1 in BALF (B) and serum (C); KC in BALF (D) and serum (E); IL-6 in BALF (F) and serum (G); and TNF-α in BALF (H). Data represent n = 3 or 4 mice per condition from one experiment and were analyzed using one-way ANOVA and Dunnett’s multiple comparisons test. *: p ≤ 0.05.
(TIF)
