# Supplementary figures and images for: Synthetic host defense peptide IDR-1002 reduces inflammation in Pseudomonas aeruginosa lung infection
Source: PLoS One. 2017 Nov 6;12(11):e0187565. doi: 10.1371/journal.pone.0187565 (PMC5673212; doi:10.1371/journal.pone.0187565)

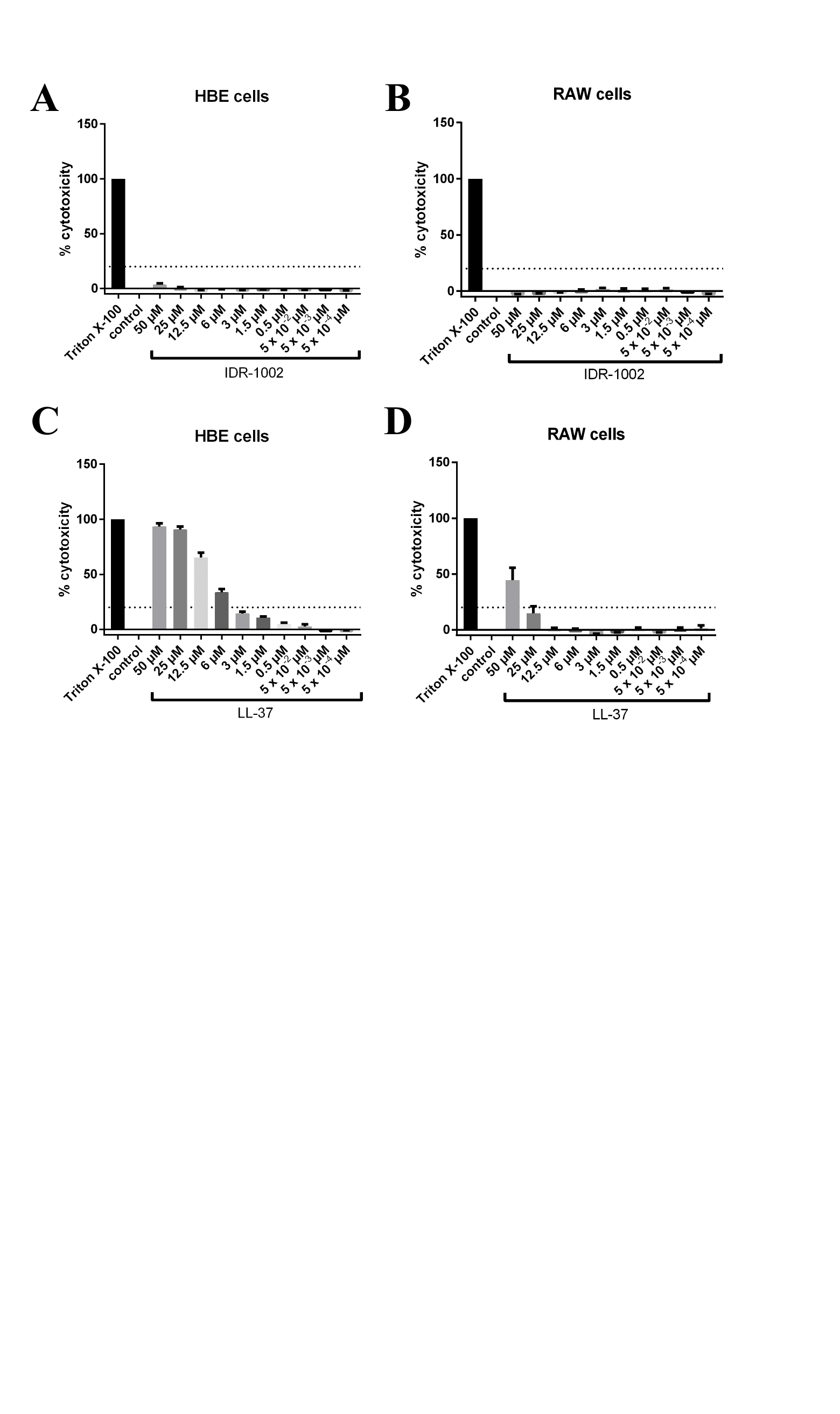

Supplement: S1 Fig — The listed concentrations of IDR-1002 (A, B) or LL-37 (C, D) were added to cells, then supernatants were collected after 24 h and used in an LDH cytotoxicity assay. The horizontal line represents 20% cytotoxicity. Data represent mean ± SEM from four (HBE cells) or five (RAW cells) independent experiments. (TIF) [file pone.0187565.s001.tif]

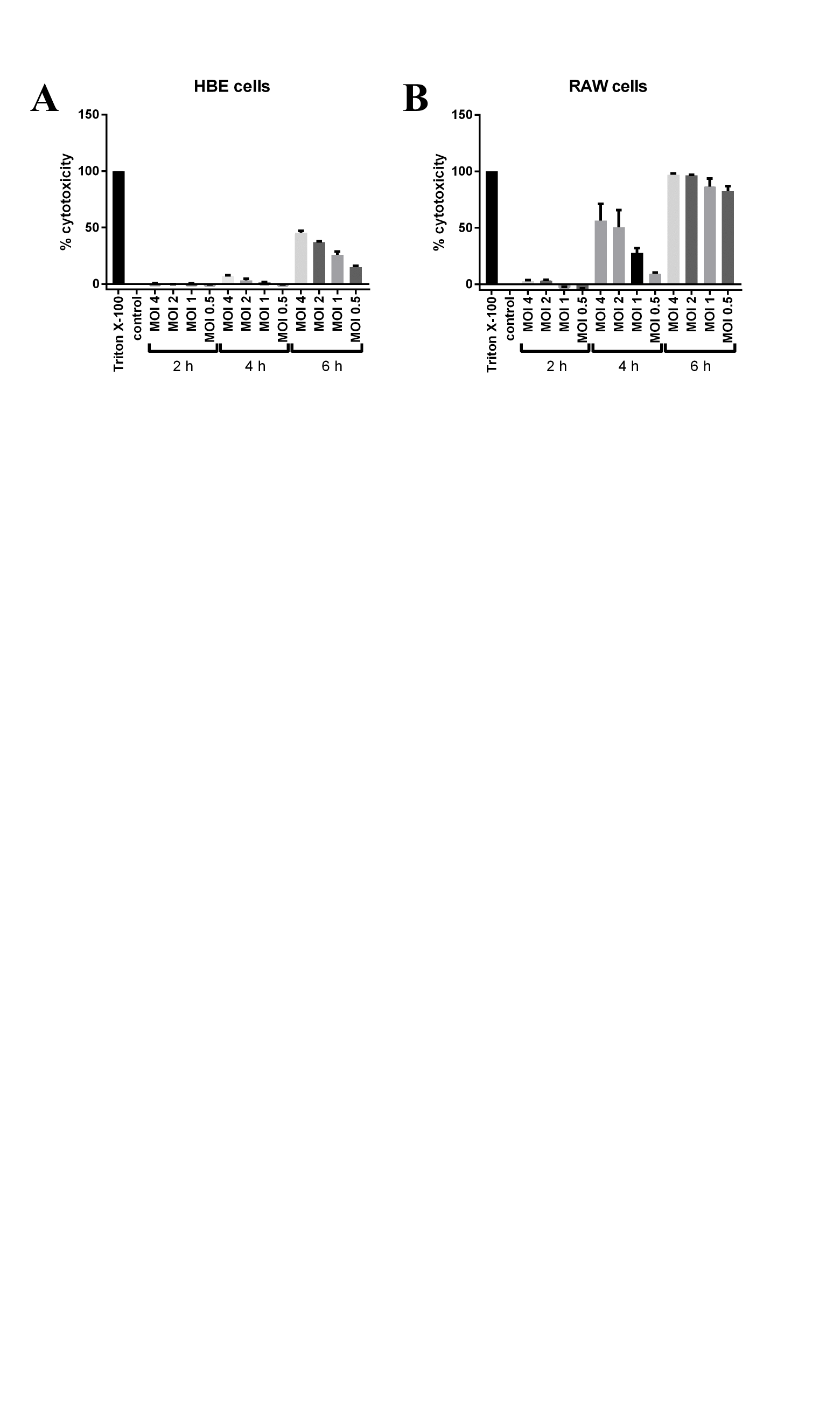

Supplement: S2 Fig — Live P. aeruginosa PA103 at MOIs of 0.5, 1, 2, or 4 was added to HBE cells (A) or RAW cells (B) at time 0 h at the same time as control and Triton X-100 samples. Samples were collected at 2, 4, or 6 h and compared to a control and Triton X-100 for each time point in an LDH cytotoxicity assay. (TIF) [file pone.0187565.s002.tif]

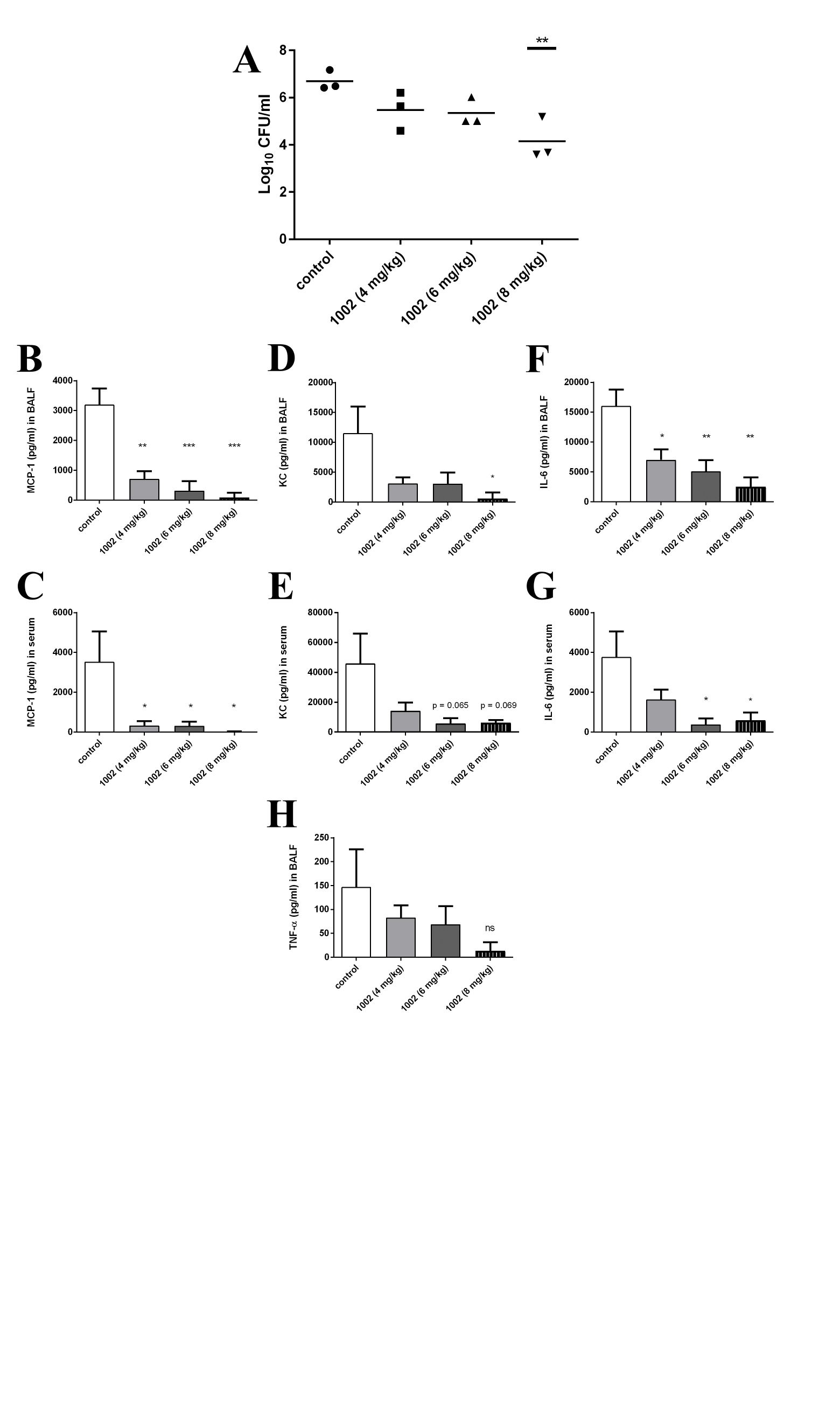

Supplement: S3 Fig — Mice were given water or IDR-1002 (4, 6, or 8 mg/kg) IN at -24 h, given 8 x 10^5^ CFUs of P. aeruginosa PA103 IN at 0 h, then euthanized at 18 h. (A) CFU counts from the BALF. ELISAs were performed for MCP-1 in BALF (B) and serum (C); KC in BALF (D) and serum (E); IL-6 in BALF (F) and serum (G); and TNF-α in BALF (H). Data represent n = 3 mice per condition from one experiment and were analyzed using one-way ANOVA and Dunnett’s multiple comparisons test. *: p ≤ 0.05, **: p ≤ 0.01, ***: p ≤ 0.001. (TIF) [file pone.0187565.s003.tif]

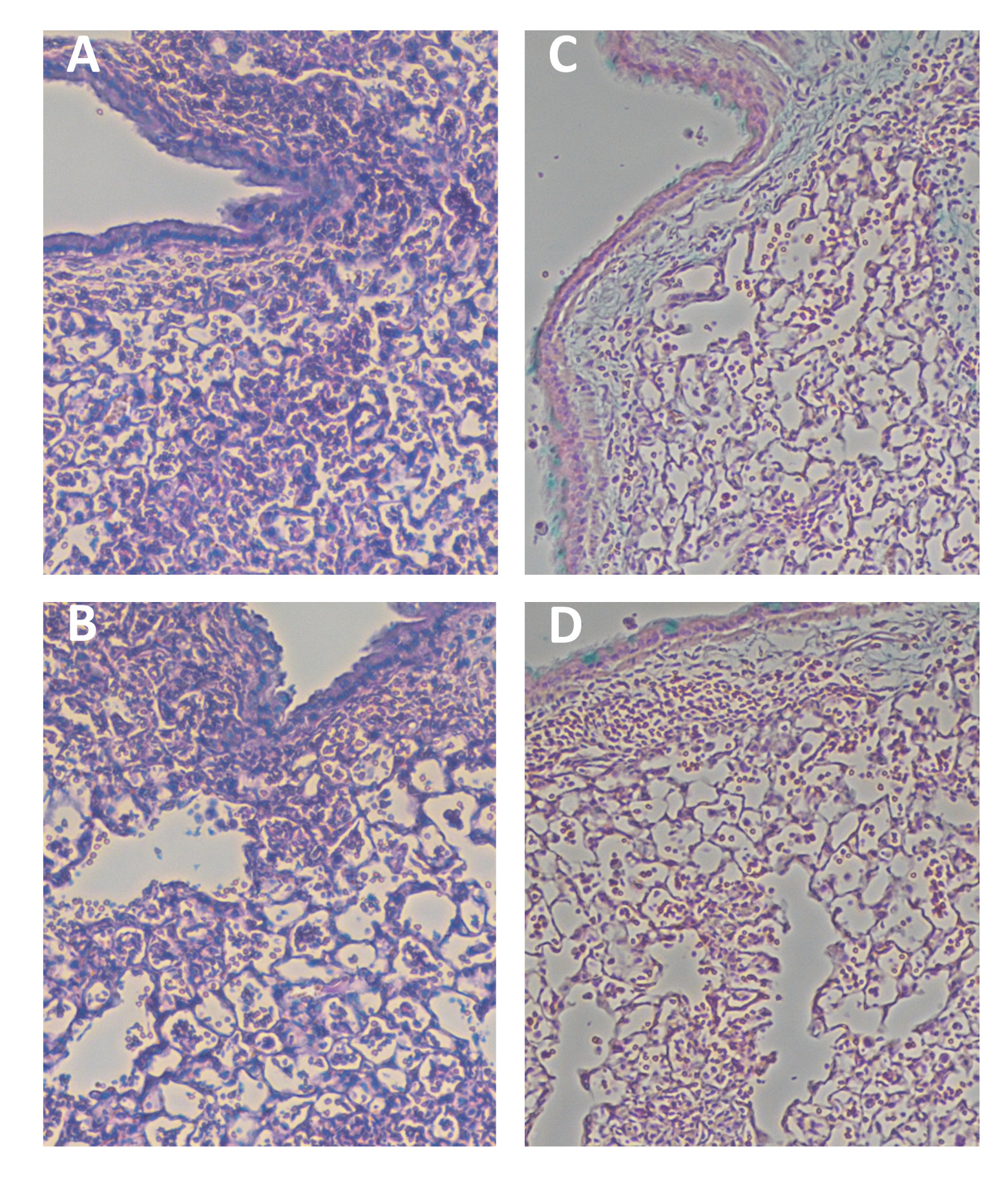

Supplement: S4 Fig — Mice were given alginate mixed with P. aeruginosa LESB58 (7 x 10^6^ CFU/mouse) IN at 0 h, then IDR-1002 (12 mg/kg) or control IN at 18 h, then euthanized at 42 h. PAS staining in the control mice (A) and IDR-1002 mice (B) and Alcian blue staining in the control mice (C) and IDR-1002 mice (D) appeared similar between the two groups. (TIF) [file pone.0187565.s004.tif]

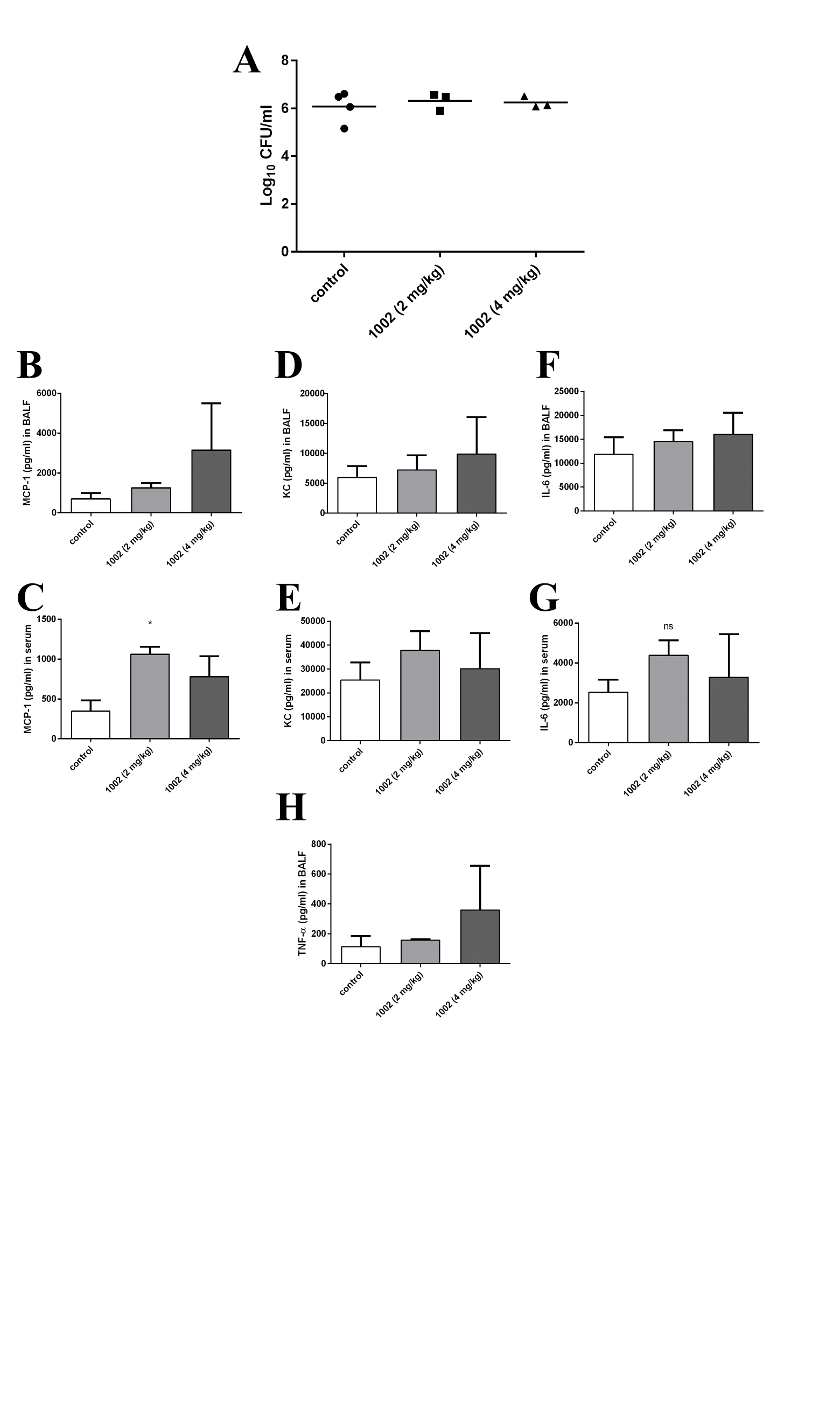

Supplement: S5 Fig — Mice were injected IP with saline or IDR-1002 (2 or 4 mg/kg) at -4 h, instilled IN with 4 x 10^5^ CFUs of P. aeruginosa PA103 at 0 h, then euthanized and samples processed at 18 h. (A) CFU counts from the BALF. ELISAs were performed for MCP-1 in BALF (B) and serum (C); KC in BALF (D) and serum (E); IL-6 in BALF (F) and serum (G); and TNF-α in BALF (H). Data represent n = 3 or 4 mice per condition from one experiment and were analyzed using one-way ANOVA and Dunnett’s multiple comparisons test. *: p ≤ 0.05. (TIF) [file pone.0187565.s005.tif]
